# Induction of the Estrogenic Marker Calbindn-D_9k_ by Octamethylcyclotetrasiloxane

**DOI:** 10.3390/ijerph121114610

**Published:** 2015-11-17

**Authors:** Dongoh Lee, Changhwan Ahn, Beum-Soo An, Eui-Bae Jeung

**Affiliations:** 1 Laboratory of Veterinary Biochemistry and Molecular Biology, College of Veterinary Medicine, Chungbuk National University, Cheongju, Chungbuk 362-763, Korea; E-Mail: eastdaylight@gmail.com; 2 Department of Biomaterials Science, College of National Resources & Life Science, Pusan National University, Miryang, Gyeongsangnam-do 627-706, Korea; E-Mail: anbs@pusan.ac.kr

**Keywords:** calbindin-D_9k_, endocrine disruptors, GH3 cell, octamethylcyclotetrasiloxane, uterotrophic assay

## Abstract

Interrupting the hormonal balance of an organism by interfering with hormones and their target receptors gives rise to various problems such as developmental disorders. Collectively, these reagents are known as endocrine disruptors (EDs). Cyclic volatile methyl siloxanes (cVMSs) are a group of silicone polymers that including octamethylcyclotetrasiloxane (D4). In the present study, we examined the estrogenicity of D4 through *in vitro* and *in vivo* assays that employed calcium-binding protein 9K (calbindin-D_9k_; CaBP-9K) as a biomarker. For *in vitro* investigation, GH3 rat pituitary cells were exposed to vehicle, 17β-estradiol (E2), or D4 with/without ICI 182 780 (ICI). CaBP-9K and progesterone receptor (PR) both were up-regulated by E2 and D4 which were completely blocked by ICI. Transcription of estrogen receptor α (ER α) was decreased by E2 and D4 but increased by ICI. D4 was also administered to immature female rats for an uterotrophic (UT) assay and detection of CaBP-9K. Ethinyl estradiol (EE) or D4 was administered subcutaneously with or without ICI. Although uterine weight was not significant altered by D4, an effect thought to be due to cytochrome P450 (CYP), it induced CaBP-9K and PR gene expression. Based on these results we reveal that D4 has estrogenic potential proven under *in vitro* and *in vivo* experimental conditions.

## 1. Introduction

Interfering with the physiologic endocrine system of an organism can lead to serious problems. It has been noted that the incidence of abortions was increased in sheep eating some clover species due to the presence of compounds that interfere with the interaction between hormones and their target receptors [1,2]. Some natural compounds with this effect are well known and most of them are limited to existing in Nature. However, novel chemicals with these properties have been developed as a result of industrialization. Diethylstilbestrol (DES) was used to reduce adverse pregnancy outcomes or as an anti-cancer reagent. In addition to these positive uses, the estrogenic effects of this compound can also induce several disorders and cancer in the fetus [3]. Nowadays, the toxicity of drugs for human consumption is verified using *in vitro* and *in vivo* experiments as well as clinical trials. Compounds produced for daily use are less stringently evaluated compared to pharmaceuticals. Although these materials are not directly absorbed by an organism, they can have potent indirect effects. Many synthetic compounds such as plasticizers or other brominated flame retardants have been defined as endocrine disruptors (EDs) that generally have estrogenic activity in humans [4]. Traditionally recognized roles of EDs are feminizing or masculinizing of the opposite sex and infertility [5,6]. More recently noted effects of EDs include reproductive epigenetic effects that alter the psychological activity of offspring [7]. Polycystic ovary syndrome (PCOS) has also been shown to be related to EDs [8]. Due to their estrogen-disrupting properties, some chemicals are now banned in various nations. Subsequently, newly synthesized chemicals are produced and evaluated to replace the banned reagents, however evidence is increasing for the notion that these chemicals have harmful cumulative characteristics though their potency is minimal [2]. Not only estrogen but some other chemicals are known to interrupt several endocrine systems. For instance, bisphenol A (BPA) is a potent estrogen receptor (ER) agonist, while it also antagonizes the thyroid receptor [9]. Likewise, EDs in daily products affect the endocrine system through various pathways and cumulative continuous exposure.

Validated biomarkers for estrogenic activity have been found in different tissues and cell types. Currently some well-known biomarkers for estrogenicity are complement component 3 (C3), vitellogenin (VTG), and CaBP-9K [10,11,12]. However, compared to other genes, the induction of CaBP-9k by E2 and EDs is stronger [13]. CaBP-9K contains an EF hand structural domain that is a calcium binding site believed to interact with calcium ions in the cytoplasm [14]. *In vitro* exposure of rat pituitary gland cells to BPA increases CaBP-9K levels [15]. In this study, CaBP-9k expression as a biomarker was sensitive enough to detect BPA at a dose of 10^−9^ M in a dose dependent manner. Other known estrogenic chemicals such as 4-*tert*-octylphenol and nonylphenol also successfully up-regulate CaBP-9K gene expression *in vivo* and *in vitro* [15].

Silicones are a complex of siloxane monomers and various forms are made up of different types of monomers, including cyclic volatile methyl siloxanes (cVMSs) that are a cyclic form of siloxane monomers. The cVMSs are found in hair and skin care products (personal care products; PCPs), sealants, and cosmetics; and are used as antiperspirants or defoamers due to their thermostability and inert characteristics [16,17]. These compounds are categorized according to the number of silicon atoms in their ring structure: hexamethylcyclotrisiloxane (D3), octamethylcyclotetrosiloxane (D4), decamethylcyclopentasiloxane (D5), and dodecamethylcyclohexasiloxane (D6). Among these, D4 is a suspected ED due to its estrogenic properties [18,19].

The CYP family consists of many subfamilies, including members with substrate-specific activity [20]. Ingestion of ethanol induces cytochrome P450, family 2, subfamily E, polypeptide 1 (CYP 2E1) protein in the liver so an organism will oxidize ethanol more rapidly and efficiently [21]. Another well-known inducer of CYP is barbiturates, that are quickly eliminated by increased CYP levels [22]. Xenoestrogens also augment the expression of some CYP family members in the liver, thus increasing the elimination rate of the compounds [23]. In a similar way, the expression of cytochrome P450, family 2, subfamily b, polypeptide 1 (CYP2B1) is elevated by administration of D4 in a dose-dependent manner so that D4 is oxidized and removed from body more rapidly [24].

In the present study, we evaluated the estrogenic effect of D4 on GH3 rat pituitary gland cells by measuring the expression levels of CaBP-9K, a well-established biomarker for estrogenicity. An uterotrophic (UT) assay was also performed following the Organization for Economic Co-operation and Development (OECD) guide for standard comparison and drug administration route.

## 2. Experimental Section

### 2.1. Chemicals

E2, EE, and D4 were purchased from Sigma-Aldrich (St. Louis, MO, USA) and ICI 182, 780 was obtained from Tocris Bioscience (Bristol, UK). Stock solutions were made by dissolving in dimethyl sulfoxide (DMSO; Santa Cruz Biotechnology, Santa Cruz, CA, USA) and diluted with media or corn oil (Sigma-Aldrich) as needed.

### 2.2. GH3 Cells

GH3 cells (ATCC, Manassas, VA, USA) were maintained in Dulbecco’s modified Eagle’s medium (DMEM; Life Technologies, Carlsbad, CA, USA) supplemented with 10% fetal bovine serum (FBS; Biowest, Nuaillé, France), 100 IU/mL penicillin, and 100 μg/mL streptomycin (Biowest), and incubated in a humidified atmosphere with 95% O_2_, 5% CO_2_ at 37 °C. The cells were then seeded in a 6-well plate at a density of 3 × 10^6^. The medium was changed to phenol red-free DMEM (Sigma-Aldrich) containing charcoal dextran-treated FBS (Biowest) 1 day after seeding. The medium was then changed every 2 or 3 days. Cell confluence reached 70%–80% after 7 days. E2 (1.0 × 10^−9^ M) or D4 (1.0 × 10^−5^ M) was administered to the cells for 1 day. ICI (1.0 × 10^−7^ M) was also added to the cells 30 min prior to treatment with E2 or D4. The final DMSO concentration in media was set at 0.1% for all compounds including vehicle. Each experiment was performed triplicate.

### 2.3. Animals

Post-natal day (PND)11 female Sprague Dawley (SD) rats with a dam were purchased from SAMTAKO (Gyeonggi-do, South Korea) and allowed to acclimate for 7 days. Each group of five pups was bred using one dam in a polycarbonate cage with non-phytoestrogen beta chip bedding. To eliminate xenoestrogenic effects except for those exerted by the drugs administered, the non-steroid pellet diet AIN-76A (Central Lab. Animal Inc., Seoul, South Korea) and sterile water were provided *ad libitum*. Temperature of the environment was set at 20–24 °C with 40%–60% relative humidity and a 12-h light-dark cycle. EE (3 μg/kg) and D4 (500 or 1000 mg/kg) were administered at PND 18 days immature rats subcutaneously for 4 days. ICI (3 mg/kg) was injected 30 min prior to delivering EE or D4. The animals were sacrificed 1 day after the last dosage. Institutional Animal Care and Use Committee (IACCUC) of Chungbuk National University approved all animal experimental procedures (Approval N. CBNUA-801-15-01).

### 2.4. UT Assay and Liver/Body Weight Ratio Measurement

Rats were anesthetized with isoflurane and weighed. The wet uterus and liver were dissected after all blood was drained from the rats and weighed. The uterus and liver weight was divided by body weight and normalized to relative to the vehicle-treated animals.

### 2.5. Quantitative Real-Time qPCR

Cells were washed twice with cold sterile PBS and lysed with TRIzol (Life Technologies, Carlsbad, CA, USA). Organs were washed with cold sterile saline and homogenized in TRIzol with a bullet blender (Next Advance, Averill Park, NY, USA). Total RNA was extracted from the homogenate according to the manufacturer’s instructions. Intensity of total RNA was confirmed by the 28S/18S rRNA ratio and 5S rRNA integrity with electrophoresis. Total RNA concentration was measured, and 1 g of RNA was transcribed using Moloney murine leukemia virus (mMLV) reverse transcriptase (iNtRON Bio, Gyeonggi-do, South Korea) with random a 9-mer primer (TaKaRa Bio Inc., Kusatsu, Japan) to produce first-strand complementary DNA (cDNA). Each amplicon (1 μL) was assayed using SYBR PCR (TaKaRa Bio Inc.) qPCR according to the manufacturer’s protocol. qPCR was performed under the following conditions: 40 cycles of denaturation at 95 °C for 30 s, annealing at 60 °C for 30 s, and extension at 72 °C for 30 s. The threshold cycle (CT) value was determined automatically at the exponential phase of the delta CT fluorescence detection graph. 18S RNA (18S) was used as an endogenous reference while CaBP-9K, ER α, PR and CYP2B1 were the genes of interest (GOI). Quantification of the GOI relative expression was calculated with respect to 18S according to the 2^−ΔΔCT^ method and normalized to vehicle.

### 2.6. Western Blotting

Cells were washed twice with cold sterile PBS and lysed with radioimmunoprecipitation assay (RIPA) buffer containing proteinase inhibitor cocktail (Sigma Aldrich). The washed organs were homogenized in Pro-prep (iNtRON) with a bullet blender (Next Advance). The homogenate or lysate was cleared by centrifugation at 18,000 G for 10 min at 4 °C. Next, 30 μg of sample were treated by mixing with SDS sample buffer, heating at 95 °C for 5 min, and centrifuging 15,000 G at 4 °C for 10 min. Afterwards, 7.5% and modified 20% SDS acrylamide was prepared and electrophoresis was performed. The separated proteins were transferred to polyvinylidene fluoride (PVDF) membrane (Merck Millipore, Taunton, MA, USA). The membrane was blocked with 5% skim milk dissolved in TBS-T for 2 h. The membrane was then incubated for 2 h with primary antibodies specific for CaBP-9K, ER α, and PR (Santa Cruz Biotechnology) diluted 1000-fold in 1% BSA. Next, the membrane was washed four times for 15 min each with TBS-T. The blot was subsequently incubated for 1 h with secondary antibody conjugated with horseradish peroxidase (rabbit polyclonal, Santa Cruz Biotechnology) diluted 3000-fold in 2.5% non-fat milk dissolved in TBS-T. The membrane was washed again as previously mentioned. Enhanced chemiluminescence (ECL) reagent (Santa Cruz Biotechnology) with a charge-coupled device (CCD) was used to detect antibody binding.

### 2.7. Statistical Analyses

All data are presented as the mean ± standard deviation (SD) and were analyzed with a one-way ANOVA and Tukey’s studentized range test. Statistical was analyses were performed with SAS (version 9.2; Chicago, IL, USA). *p*-values less than 0.05 were considered significant.

## 3. Results

### 3.1. CaBP-9K, ER α, and PR Expression in GH3 Cells

**Figure 1:**
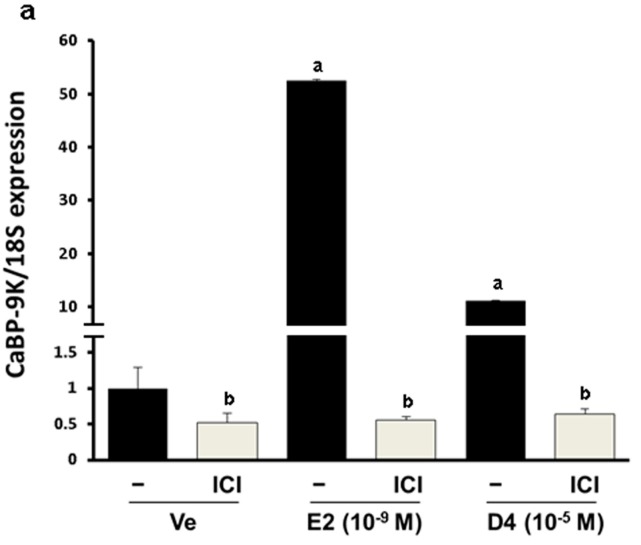

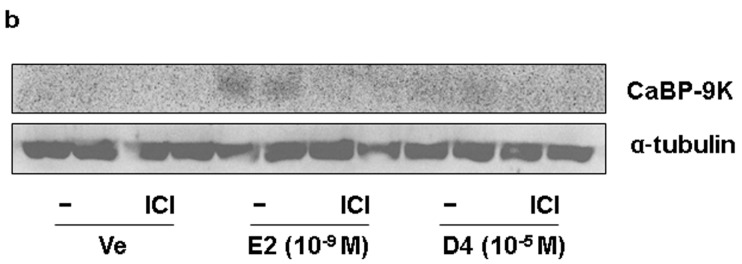
CaBP-9K expression in GH3 cells at the mRNA (**a**); and protein (**b**) levels. Results presented in the bar graph are divided according to chemical administered and subdivided according to the presence or absence of ICI 182 780. Ve, vehicle; E2, 17β-estradiol; D4, octamethylcyclotetrasiloxane. **^a^** *p* < 0.05 *vs.* vehicle; **^b^** *p* < 0.05 *vs.* without ICI. Data are presented as the mean ± SD.

**Figure 2:**
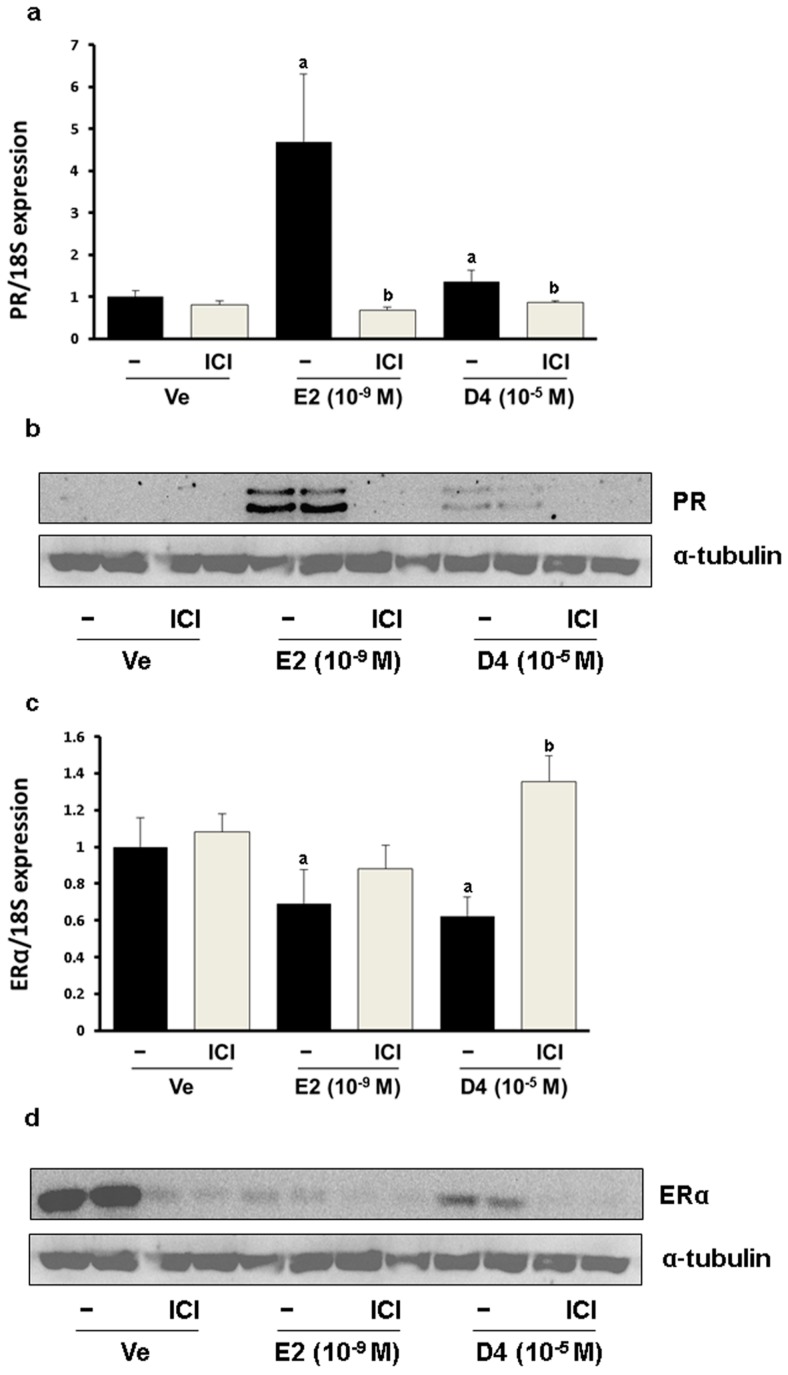
PR expression in GH3 cells at the mRNA (**a**); and protein (**b**) levels; ER α expression in GH3 cells at the mRNA (**c**); and protein (**d**) levels. Results presented in the bar graph are divided according to chemical administered and subdivided according to the presence or absence of ICI 182 780 (shown as ICI). **^a^** *p* < 0.05 *versus* vehicle; **^b^** *p* < 0.05 *versus* without ICI. Data are presented as the mean ± SD.

To verify the estrogenic effects of D4, CaBP-9K gene expression was examined in GH3 cells. The GH3 cell line is derived from rat pituitary and expresses ER, PR and CaBP-9k, therefore it is a suitable *in vitro* model to test estrogenic effects. In the real-time PCR results, relative mRNA expression levels of CaBP-9K in the E2- and D4-treated groups were increased compared to the vehicle control (Figure 1a). To evaluate the pathway of gene expression response to D4, ICI that is a well-known ER antagonist and uses to block ER-mediated signaling, was treated together. ICI decreased the induction of CaBP-9K expression in all groups. Protein expression of CaBP-9K was similar to the mRNA levels, although the basal expression levels of CaBP-9k was lower by showing faint band (Figure 1b). Another well-known estrogen target gene, PR, was also examined in this study (Figure 2). Both mRNA and protein levels of PR were significantly enhanced by E2 and D4, which was completely blocked by ICI (Figure 2a,b). In contrast, ER α mRNA and protein expressions were down-regulated by E2 and D4; ICI reversed this effect (Figure 2c,d).

### 3.2. Relative Weight Ratio and CYP2B1/2 Expression in Immature Rat Liver

**Figure 3:**
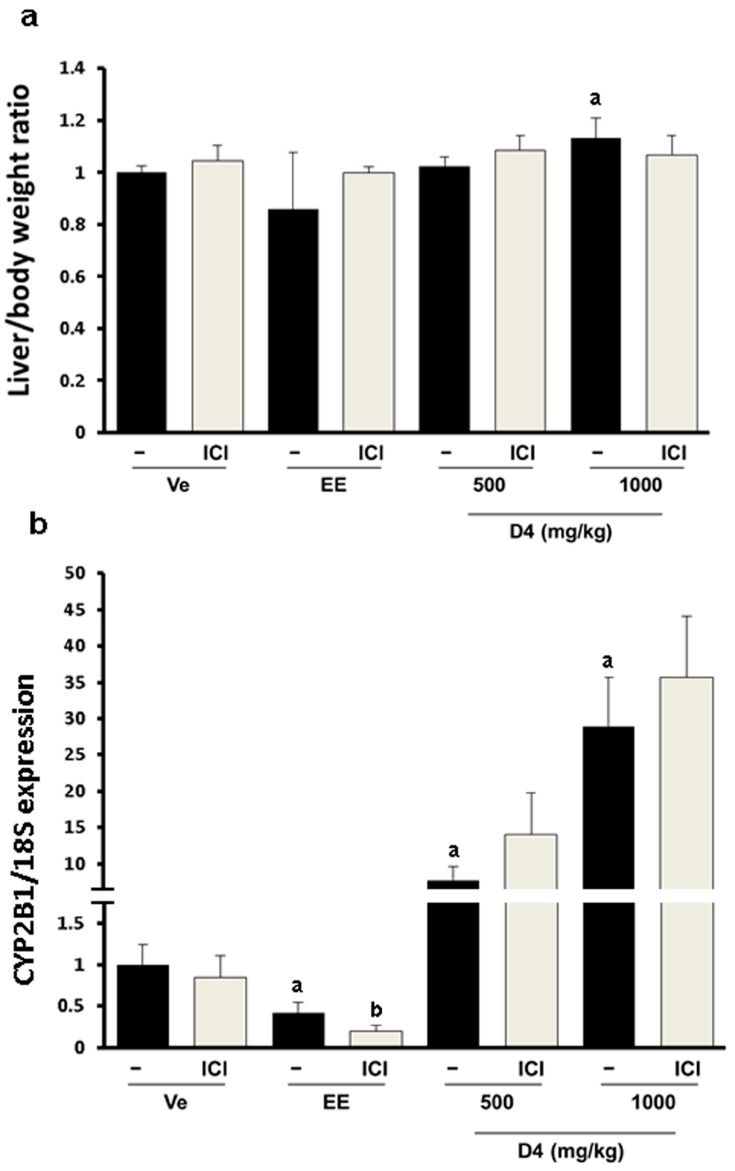
Liver/body weight ratio (**a**); and CYP2B1 mRNA expression (**b**) in immature female SD rats. Data presented in the bar graph are divided according to chemical administered and subdivided according to the presence or absence of ICI 182 780 (shown as ICI). **^a^** *p* < 0.05 *vs.* vehicle; **^b^** *p* < 0.05 *vs.* without ICI. Data are presented as the mean ± SD.

To examine the toxicological effects of D4 *in vivo*, female immature rats were administrated with EE and D4 (500 and 1000 mg/kg) for four days. Liver/body weight ratios were not changed in the EE-treated group compared to the vehicle group, whereas they were slightly but significantly increased in the D4-animals treated at a high dosage. This ratio was unaltered in groups co-treated with ICI compared to the rats receiving EE or D4 alone (Figure 3a). CYP2B1/2 mRNA expression was highly induced by D4 in a dose dependent manner, while it was reduced by EE. ICI had no effect or slightly increased the induction of CYP2B1/2 expression in the D4 treatment group. EE co-treatment with ICI also had no effect or slightly decreased the expression of CYP2B1/2 (Figure 3b).

### 3.3. UT Assay Results in Immature Rats

**Figure 4:**
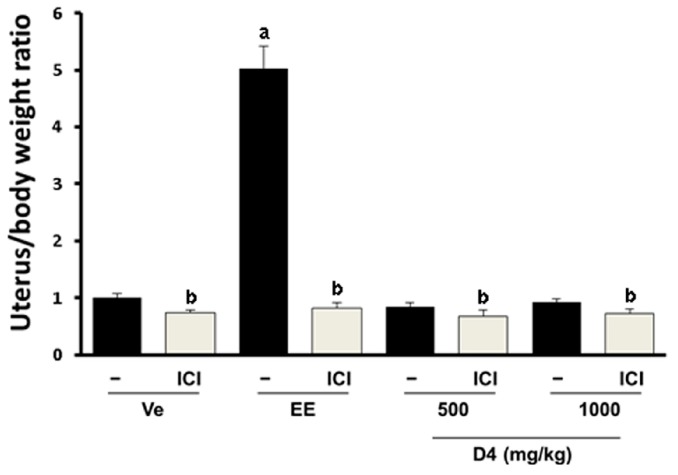
Uterus/body weight ratio measured by the UT assay in immature female SD rats. Results in the graph are divided according to the chemical administered and subdivided according to the presence/absence of ICI 182 780 (shown as ICI). **^a^** *p* < 0.05 *vs.* vehicle; **^b^** *p* < 0.05 *vs.* without ICI. Data are presented as the mean ± SD.

Immature rats were administrated with EE and D4, and the uterine weight was measured to confirm the estrogenic effect of D4 following the OECD guidelines. As we expected, E2 induced up to five fold uterus/body weight ratio increases, suggesting that our experimental conditions were working successfully. However, administration of rats with D4 did not show any significant changes (Figure 4). These results indicate that estrogenic effect of D4 is not strong enough when examined by the UT assay.

### 3.4. CaBP-9K, ER α, and PR Expression in Immature Rats Uterus

**Figure 5:**
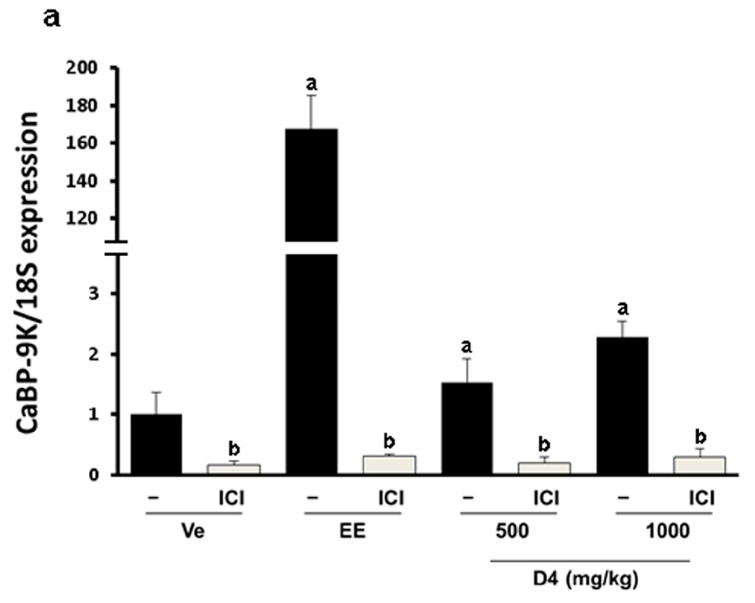

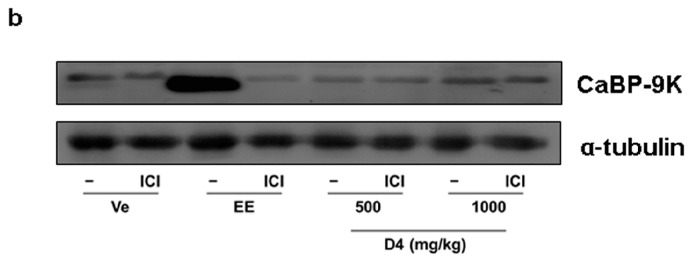
CaBP-9K expression at the mRNA (**a**); and protein (**b**) levels in immature female SD rats. Data presented in the graph are divided according to chemical administered and subdivided according to the presence or absence of ICI 182 780 (shown as ICI). **^a^** *p* < 0.05 *vs.* vehicle, **^b^** *p* < 0.05 *vs.* without ICI. Data are presented as the mean ± SD.

**Figure 6:**
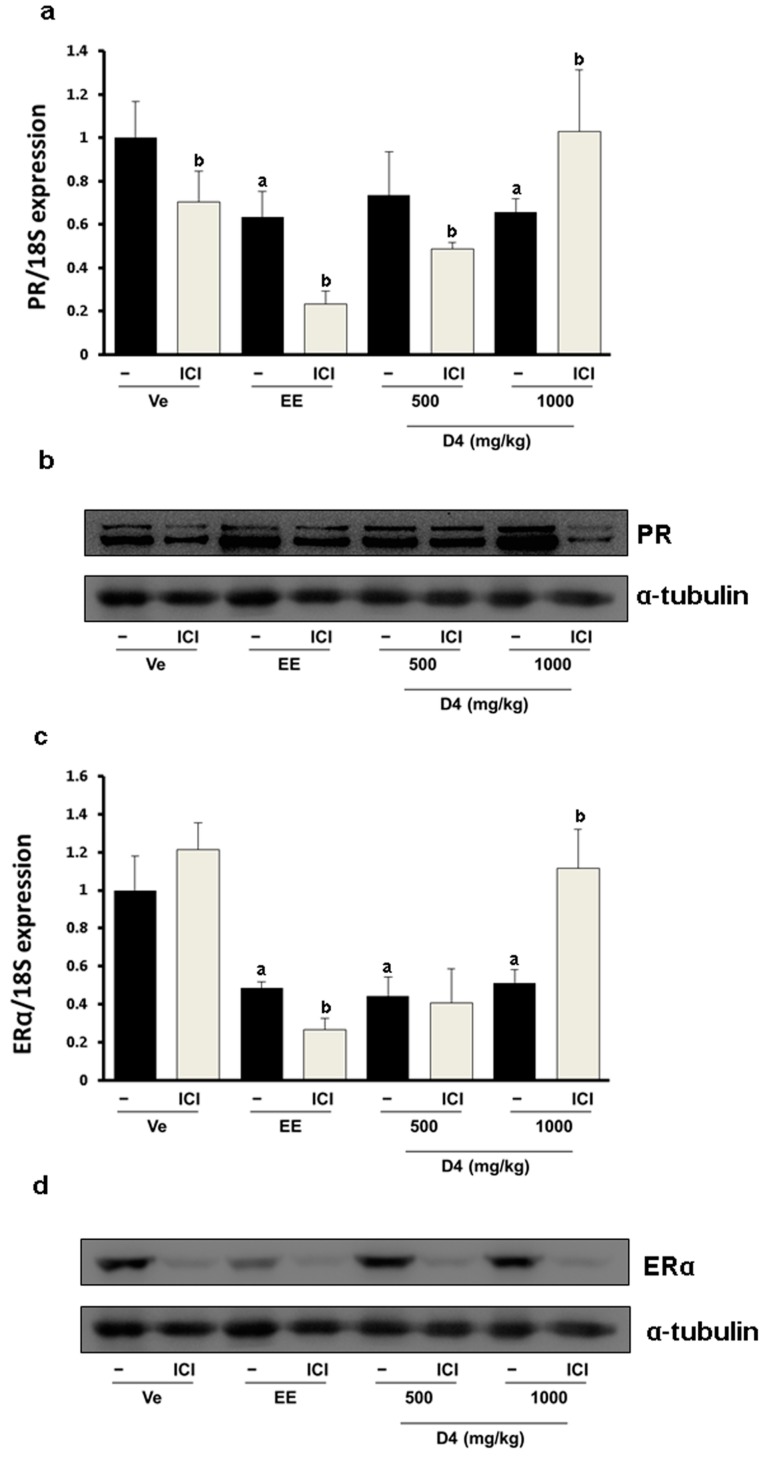
PR expression at the mRNA (**a**); and protein (**b**); levels in immature female SD rats. ER α expression in immature female SD rats at the mRNA (**c**); and protein (**d**) levels. Findings presented in the bar graph are divided according to chemical administered and subdivided according to the presence or absence of ICI 182 780 (shown as ICI). **^a^** *p* < 0.05 *versus* vehicle, **^b^** *p* < 0.05 *versus* without ICI. Data are presented as the mean ± SD.

Since the estrogenic effect of D4 was not shown by the UT assay, we used a more sensitive method. In the uterus, the estrogenic biomarker CaBP-9K mRNA expression was significantly increased by EE and D4 in a dose-dependent manner. Increases in mRNA levels were inhibited in the ICI-co-treated group (Figure 5a). The protein expression levels of CaBP-9K showed concomitant results with those of mRNA by elevating after EE. CaBP-9K mRNA expression was up-regulated about 2- or 3-fold by 500 and 1000 mg/kg of D4, while the regulation by EE was 170-folds higher. The regulation of CaBP-9k protein by EE and D4 was similar with those of mRNA. Co-treatment with ICI decreased CaBP-9K protein levels compared with EE or D4 alone (Figure 5b). Interestingly, transcriptional and translation regulation of PR by EE and D4 was different. PR mRNA expression was suppressed by EE and a high dose of D4, and ICI further decreased it, while EE and D4 increased PR (Figure 6). ER α mRNA expression was decreased by EE and D4 (Figure 6c). ICI co-treatment with EE also led to reduced ER α mRNA and protein expressions but the expression levels were not decreased in the D4 treatment group as much as the EE-exposed animals (Figure 6d).

## 4. Discussion

The hazardous effects of EDs have led to an establishment of screening tests for thousands of compounds. Many simple high-throughput screening systems (e.g., an ER-binding assay and AR binding assay) using a vector expressing hormone response elements (HREs) such as an estrogen response element (ERE) or androgen response element (ARE) have been used to measure compound potency [25]. However, these systems do not account for the physiological metabolism of an organism represented as absorption/administration, distribution, metabolism, excretion, and toxicity (ADMET). To more rapidly assess the potential of an ED, some *in silico* methods have been developed and tested based on existing databases [26]. However, not all of these tests can precisely evaluate the pharmacokinetics and pharmacodynamics model (PK/PD) of an ED. Some modified *in vitro* assays have been established to mimic these PK models specifically targeting each step. In the Caco-2 cell permeability test, the absorption of a drug in the gastrointestinal (GI) tract is mimicked [27]. The *in vitro* unbound fraction and hepatic microsome activity test is also designed to mimic renal and hepatic clearance that is part of metabolism and excretion [28]. Finally, toxicity tests are performed by receptor binding assays. To overcome systemic differences between *in vitro* and *in vivo* techniques, *in vitro* to *in vivo* extrapolation (IVIVE) has been recommended and tested along with *in vivo* systems [29,30].

Different countries or world organizations have established acceptable compound test guides for identifying the potential hazardous effects of chemicals. The Endocrine Disruptor Screening Program (EDSP) of the U.S. Environmental Protection Agency provides several basic to detailed protocols for screening drugs. The OECD has also produced many test guides for evaluating drugs. Validated assays for testing estrogenic compounds provided by the EPA include the ER binding assay. The OECD recommends the stably transfected human ER α transcriptional activation assay (ER STTA) and UT assay in rodents. Monitoring the induced expression of specific markers is another inexpensive tool for detecting hormone-like effects.

The ERE promoter is located upstream of the CaBP-9K gene transcriptional initiation site. E2 and other potential EDs bind to the ER and these are active the promoter, and initiate the expression of CaBP-9K. Therefore, CaBP-9K has been used as a sensitive marker for detecting estrogenic activity [15]. In this study, expression of CaBP-9K was highly induced by E2 and slightly increased by D4 in GH3 cells. Many previous studies have demonstrated that CaBP-9K expression is induced by various EDs both *in vitro* and *in vivo* [31,32,33], including in GH3 cells [34,35]. ICI, an ER α inhibitor, decreased CaBP-9K expression elevated by E2 and D4. Down-regulation of CaBP-9K expression following ICI exposure indicated that the weak estrogenicity of D4 may be mediated by ER α. The estrogenic effect of D4 was also shown previously by a simple ER binding affinity assay [18].

The enzyme CYP2B1 mainly participates in oxidation procedures for the elimination of drugs [36]. Also oxidation of xenobiotics by CYP contributes to inactivate the xenobiotics. In our results, enzymatic catalysis of D4 by CYP2B1 was predicted in a dose-dependent manner according to the induction of mRNA expression. This was also consistent with a high D4-treated group that showed a slight increase of liver/body weight ratio due to enzyme induction. Considering the general role of CYP in the liver, induction of CYP2B1 may be thought as metabolic process to eliminate and inactivate D4. CYP2B1 enzyme induction by D4 was previously evidenced [24]. Other CYP family proteins beyond CYP2B1 were also reported to be induced by D4 for its metabolism [37]. This xenobiotic is metabolized by the liver, the major route for the elimination of toxins or other compounds from the body [38].

Global distribution of D4 is well documented and its concentration varies from 0.66 to 45 ng/m^3^ [39]. The concentration of D4 in European cosmetics and personal care products is in the 0.18 mg/g mean and 0.00067–5 mg/g range [17]. Daily external dermal exposure of D4 is measured at 0.08 mg/day. Another article reported the concentration of D4 and its predicted dermal exposure in Canada [40]. Concentrations vary depending on product type and range from 0.01 mg/g in hair spray to 3 mg/g in antiperspirant. The human daily dermal exposure prediction model of D4 in body lotion is 0.5 mg/day and for antiperspirant it is 0.01 mg/day. The D4 concentration in food related with silicone use ranges from 0.001 to 0.306 mg/g [41]. Women with silicone gel filled implants are more vulnerable to D4 exposure and its concentration in tissue varies from 11.9 ng/g to 1333.8 ng/g, depend on tissue type [42]. Because of its high solubility in lipids, most of the D4 absorbed in the body is accumulated in fat [43,44]. Although the environmental exposure dose of D4 is much lower than our experimental conditions, the daily accumulated and chronic amount of doses is not estimated and is hard to compare. Therefore, we aimed in the present study, to test the estrogenic effects of D4 for the potential of EDS.

In the present study, ER α expression was decreased by E2 and D4 at both the mRNA and protein levels, which was also found in other studies with MCF-7 cells [45,46]. Additionally, decreased ER α expression by E2 is thought to be modulated by the ErbB2/PI 3-K/Akt pathway in GH3 cells. Dissimilar mRNA and protein expression patterns were observed when ICI was administered in GH3 cells. Characteristic degradation of ER α by ICI was observed at the protein level and has been described in the literature [47,48,49]. The mRNA expression of ER α was increased by ICI and similar changes have also been previously observed in MCF-7 cells [50]. ER α protein level was reduced by D4 in *in vitro*, but no difference was observed *in vivo*. This difference was similar with that of CaBP-9K.

Estrogenic properties of D4 mediated by the ER α have also been reported using mouse and rat UT assays [19,51]. However, these assays were not conducted following the OECD guidelines in that the treatment period was extended or another drug administration route was used. Thus, we delivered D4 subcutaneously to immature female rats as recommended by the OECD. Most D4 contact takes place on the skin [17]. Furthermore, subcutaneous exposure occurs among women undergoing surgery for breast implant placement [42]. An inhalation test has been performed to evaluate the volatility properties of D4 [18]. We concluded that simulation of absorption through the GI tract and an inhalation test of D4 have already been performed. D4 is mainly contained in cosmetics, so subcutaneous injection was conducted considering the main route of exposure of D4 in the body. Also the OECD’s general experimental protocol for the UT assay for endocrine disruptors is assessed by subcutaneous administration. In the results, the observed effects of D4 were not strong enough in the UT assay compared to the *in vitro* findings, whereas, it successfully modulated biomarker genes such as CYP in the liver and CaBP-9k in the uterus. These results suggest that D4 shows estrogenic effects by inducing biomarker genes, but it is not strong enough to induce physiological changes in the uterus. One biomarker, PR, was differently regulated in the *in vitro* and *in vivo* experiments. *In vitro*, D4 significantly upregulated PR in mRNA and protein levels in GH3 cells. In the animal model, transcriptional and translational levels of PR in the uterus were differently regulated by D4. D4 administration reduced mRNA levels of PR, while it induced protein levels at a high dose, although this was blocked by ICI. This suggests that transcriptional PR regulation by EE and D4 may be different from the translational process. In previous studies, different regulation of PR between transcription and translation processes has been also reported in the uterus [52].

Silicones have been used widely due to their inert nature and stability under various conditions [53]. cVMSs are classified according to the number of silicon entities in their ring. D4, D5, and compounds depend on fewer or more silicon entities. These compounds have a high octanol water coefficient (Kow) indicating a preference for dissociation in lipid-rich tissues such as brain or adipose tissue in the abdomen [43]. Additionally, they have high vapor pressure and low molecular weight. Thus, these reagents are very volatile and are commonly found in the environment [39]. cVMSs have been produced in bulk in some nations that cannot ignore the effects of these compounds. Most breast implants currently used were made of silicone for safety reasons. Complexes of silicone used in implants are made from siloxane monomers. During the synthesis of silicones, many side products are also produced, including cVMSs. It has been known that D4 but not D5 has an ability to react with the ER [18]. Specific reactions of D4 with ER may be due to a slightly different morphological 3D chemical structure between cVMSs. The reactivity of D4 with ER was well established in an *in vitro* system when just ligand and receptor exist, however it was tolerated in an *in vivo* system.

## 5. Conclusions

In conclusion, we have examined the estrogenic effects of D4 *in vitro* and *in vivo*. In GH3 cells, D4 strongly induced CaBP-9k gene expression, which was diminished by ER antagonist, ICI. Although the UT assay failed to confirm the estrogenicity of D4 *in vivo*, our biomarker system was sensitive enough to reveal estrogenic effects of D4 in the uterus, suggesting that high doses of D4 possess estrogenic properties and should be used in industrial fields with caution.
